# Differences in Patterns of Reproductive Allocation between the Sexes in *Nicrophorus orbicollis*

**DOI:** 10.1371/journal.pone.0143762

**Published:** 2015-11-24

**Authors:** Ashlee N. Smith, J. Curtis Creighton, Mark C. Belk

**Affiliations:** 1 Department of Biological Sciences, Purdue University Calumet, Hammond, Indiana, United States of America; 2 Department of Biology, Brigham Young University, Provo, Utah, United States of America; Leiden University, NETHERLANDS

## Abstract

Organisms are selected to maximize lifetime reproductive success by balancing the costs of current reproduction with costs to future survival and fecundity. Males and females typically face different reproductive costs, which makes comparisons of their reproductive strategies difficult. Burying beetles provide a unique system that allows us to compare the costs of reproduction between the sexes because males and females are capable of raising offspring together or alone and carcass preparation and offspring care represent the majority of reproductive costs for both sexes. Because both sexes perform the same functions of carcass preparation and offspring care, we predict that they would experience similar costs and have similar life history patterns. In this study we assess the cost of reproduction in male *Nicrophorus orbicollis* and compare to patterns observed in females. We compare the reproductive strategies of single males and females that provided pre- and post-hatching parental care. There is a cost to reproduction for both males and females, but the sexes respond to these costs differently. Females match brood size with carcass size, and thus maximize the lifetime number of offspring on a given size carcass. Males cull proportionately more offspring on all carcass sizes, and thus have a lower lifetime number of offspring compared to females. Females exhibit an adaptive reproductive strategy based on resource availability, but male reproductive strategies are not adaptive in relation to resource availability.

## Introduction

The cost of reproduction is the trade-off between current reproductive effort and future survival and reproduction [1]. Resources that are allocated to current reproduction cannot be allocated towards future survival and reproduction [2]. As a consequence, organisms must balance the fitness benefits of allocating resources to current reproduction with the potential costs to future reproductive opportunities [3–4]. Thus, the cost of reproduction can act as a constraint on the level of current parental investment for iteroparous organisms. For example, young individuals that allocate maximum available energy to current reproduction are predicted to experience decreased survival and overall lower lifetime fecundity compared to individuals that allocate less than the maximum available energy to early reproduction [3,5]. Empirical tests of the cost of reproduction and resulting patterns of reproductive allocation have been reported for females of a number of species [6]. However, there have been few attempts to test for generality of the theoretical solutions to the cost of reproduction tradeoff (viz. between sexes or among differing environments).

As a consequence of fundamental differences between the sexes and resulting sexual conflict (e.g., gamete production, parental care, etc.), the fitness benefits and the costs associated with specific patterns of reproductive allocation likely differ between the sexes [7]. Costs of reproduction in female arthropods are mainly associated with the production of eggs and parental care (e.g., [8–12]). In contrast, in male arthropods costs are typically associated with mating or sexual advertisement (e.g., [13–15]). In species where males provide parental care, there are costs associated with both mating and parental care (i.e. [9,16]).

How does one evaluate sex-specific life history traits in species that provide biparental care? The standard approach of manipulating brood size may be limited because the level of investment in biparental species is predicted to be, in part, a result of negotiated investment between the sexes. If the sexes are not affected by the manipulation in the same way (i.e. because of differences in the relative importance of the cost of reproduction or rates of senescence), the dynamics of care may result in over- or under- estimating the relative costs. Any interpretation of the experimental results would be confounded by this complication. An alternative approach would be to simultaneously remove one sex and manipulate brood size. However, this approach also has difficulties: removing one sex potentially alters the co-evolved pattern of biparental investment, as demonstrated in many mate removal experiments, again resulting in an over- or under-estimation of the costs [17]. One approach to overcome these limitations is to focus on species where both biparental and uniparental care naturally occur within a population [18–19].

Burying beetles (genus *Nicrophorus*) provide an ideal model system for comparing costs of reproduction and patterns of allocation in a biparental system because although biparental care is most common, both male and female uniparental care also occurs [20–25]. Both sexes can separately and independently provide all pre- and postnatal parental care required to successfully raise a brood. Broods are raised on a small vertebrate carcass, which is the sole source of food for parents and offspring during a reproductive attempt [22]. As a result, carcass mass that is consumed by the parents is an investment in future offspring because that resource cannot be allocated to the current brood if resource availability constrains reproduction.

Burying beetle parental care consists of preparing the carcass by burying it and then removing hair, rolling the carcass into a ball, and applying oral and anal secretions that prevent decay. Once the young arrive on the carcass, parents defend the carcass and larvae and feed the offspring [20,26]. The eggs are laid in the soil around the carcass, and in *Nicrophorus orbicollis* the larvae arrive on the carcass 5–7 days after the parents arrive on the carcass. The larvae feed directly on the carcass through a small hole made by the parents, and also receive regurgitate derived from the carcass from both parents [27]. Parents use carcass volume to determine carcass size [28]. Both parents use this proximate cue to regulate brood size to match carcass size through culling of offspring [24,29–30], which results in a positive correlation between offspring number and carcass size [29–31].

In this study, we compare costs of reproduction and patterns of reproductive allocation between uniparental male and female burying beetles (*N*. *orbicollis*). Specifically, we (i) manipulated reproductive resources of breeding males and females to evaluate the relative importance of the cost of reproduction as a constraint in the two sexes; (ii) compared life time fitness of males and females in terms of lifespan, and number and size of offspring produced, and (iii) evaluated the change in resource allocation between current and future reproduction as males and females aged.

## Methods

### Source of Burying Beetles

Burying beetles used to generate the laboratory-born population for our experiments were captured at the same site in central Wisconsin during summers from 2006 to 2011 using pitfall traps baited with aged chicken. These beetles were caught on private land, and permission to collect beetles was obtained from the owners. No endangered or protected species were collected. Wild-caught pairs were placed on 30-g mouse carcasses and allowed to breed to generate the laboratory population. The date of eclosion was recorded for all first generation laboratory-bred beetles. They were placed individually in small plastic containers (15.6 x 11.6 x 6.7cm) with *ad libitum* raw chicken liver and maintained on a 14:10h light:dark cycle until their use in the experiments as detailed below. The experiment for the female uniparental treatments was conducted in 2007 and 2008, and these data were previously used in a study of female costs of reproduction by Creighton et al. [5]. We conducted experiments for the male uniparental treatments during 2010 and 2011, and it is these experiments that we describe in this study.

To compare costs of reproduction between males and females we combined data from the male uniparental treatments derived from these later experiments with data from the previously published study on female uniparental treatments for analysis. Although there is a temporal difference in the experiments, all individuals were collected from the same site and were subjected to the same experimental conditions, procedures, and treatments. Further, during the time frame of the current study, two experiments evaluating female life history were performed with beetles from this population. Both experiments produced similar results in regard to female life history patterns [5,32]. Thus, temporal variation in female life history (in the context of our experiment) seem to be minimal, allowing us to compare male and female life histories with confidence.

### Experimental Design

#### Parental Care Experiment

The purpose of this experiment was twofold. First, we evaluated the change in parental investment pattern of males as they aged. Second, we manipulated resource availability as described below to evaluate the cost of reproduction hypothesis. The experimental design followed that used for females in the previous study [5] with the exception that we added an under-allocation treatment for both sexes.

We manipulated resource levels during reproduction to create conditions as if parent burying beetles overinvested (by exchanging a large carcass with a small carcass after the young arrive on a carcass) or underinvested (by exchanging a small carcass with a large carcass) in current offspring. Parents assess carcass size during preparation, and subsequently cull offspring based on this assessment. When carcasses are switched immediately after young arrive, the number of young culled by the parents reflects the original carcass size and not the substituted carcass size. For example, female *N*. *orbicollis* initially given a 30-g carcass that is subsequently replaced by a 20-g carcass as the larvae first begin to arrive (before culling is completed) rear the number of young typically raised on a 30-g carcass and not a 20-g carcass [5]. This partitioning of assessment and culling behavior from parental care behavior during later stages allows us to indirectly manipulate brood size without the parents responding to the manipulation [5]. As a result, we are able to assess the effects of the cost of reproduction independent of confounding issues created by brood augmentation or physiological manipulation [5].

The experimental design consisted of four treatments: 20-g control, 30-g control, 30-g → 20-g experimental, and 20-g → 30-g experimental. In each of the control treatments, the original carcass was left with the parent for the duration of the trial. In the 30-g → 20-g experimental treatment the 30-g carcass was replaced with a pre-prepared 20-g carcass within 12 hours of the arrival of larvae on the carcass. This treatment allowed us to determine the beetle’s responses to overproduction of offspring compared to the available resources. In the 20-g → 30-g experimental treatment the 20-g carcass was replaced with a pre-prepared 30 g carcass within 12 hours of the arrival of larvae on the carcass. This treatment allowed us to determine the beetle’s responses to underproduction of offspring compared to the available resources. Although carcasses were switched in the experimental treatments, carcasses were not switched in the control treatments because previous research found that switching a carcass with one that was pre-prepared of the same size had no effect on reproduction [5]. In addition, each brood was handled daily, regardless of treatment to count larvae and check brood progress. Therefore, control carcasses were not replaced entirely but were removed briefly and replaced in a similar way to the experimental treatments. In all treatments, containers were kept the same. Twelve replicates were completed for each of the four treatments for males for comparison to the previously documented patterns of females [5]. Combining data from males from this experiment (plus females in the underallocation treatment) and females from the Creighton et al. [5] study resulted in 96 total replicates.

The experimental protocol consisted of measuring and recording traits of individual parents and offspring for each reproductive bout during the parents’ entire lifetimes. The mass, pronotum width, and date of eclosion were recorded for each individual that was used for a trial. Pronotum width correlates strongly with overall body size (a potentially useful covariate). To begin each trial we randomly chose a genetically unrelated, 28 day old, virgin male and female to match the protocol used by Creighton et al. [5]. We randomly assigned each pair to one of the four treatments, and individuals were assigned to the same treatment for the duration of their lifetime. The pair was placed in a small brood container (16.5 x 15 x 9 cm) filled with 6cm of moist soil and either a 20-g (± 1.0-g) or 30-g (± 1.0-g) mouse carcass, depending on the treatment. The containers were kept in an environmental chamber at 21°C on a 14:10 h light:dark cycle to simulate breeding conditions in August. After the larvae hatched, the female was removed, and the remaining male provided parental care alone. When the larvae dispersed into the soil, the male was weighed and placed on *ad libitum* chicken liver, then placed on a new carcass of the same size two days later with a genetically unrelated female that had not previously mated. The cycle continued for the focal male parent beetle until death. The larvae from each brood eclosed 4–5 weeks after dispersal. On the day of eclosion for each individual offspring, the date was recorded, along with the mass and pronotum width of each individual. The experimental protocol for females in the underallocation treatment was the same as that described for all other treatments.

#### Non-Breeding Experiment

The purpose of this experiment was to determine the life spans of males when they did not reproduce for comparison to females from the previous study. Twenty-four males were selected and placed on *ad libitum* chicken liver for the duration of their life spans. Date of eclosion and date of death were used to determine life span of each individual.

### Statistical Analyses

#### Lifetime Fitness Measures

To determine differences in lifetime fitness between males and females and among treatments, we combined data from the above experiments on males with data on females from Creighton et al. [5] and additional data from females for the underallocation treatment. For summary lifetime measures of fitness, we used two response variables: life span and lifetime number of offspring. The experiment was designed as a fully crossed factorial design with two fixed factors: sex (2 levels) and carcass treatment (4 or 5 levels). We evaluated the two response variables for normality of residuals and equal variances across treatment combinations. The data met these assumptions. We also tested size (pronotum width) of individuals as a covariate. Size was not a significant effect in preliminary analyses and was not used in the final analysis. The analysis of life span (days from eclosion to death) included the treatment of non-reproducing individuals. For lifespan and lifetime number of offspring we used a model where sex and carcass treatment were main effects (fixed) and we included the sex by carcass treatment interaction (Proc GLM in SAS; SAS 9.3 SAS Institute, Cary, North Carolina, USA).

#### Within Lifetime Patterns

In our second set of analyses we compared within lifetime patterns of change in life history traits. We analyzed three response variables for their within lifetime pattern of change: initial brood size, final brood size, and mean individual offspring mass per reproductive bout. Initial brood size is the number of offspring on the carcass before the parent has reduced the brood through culling. This serves as a measure of the number of eggs laid on the carcass. It may seem odd to include sex as a factor in initial number of eggs laid because that is dependent on the female. However, there is potential for females to respond to male age or quality by changing the number of eggs that they lay, and there is also the possibility that males are able to produce less sperm over time and thus limit the number of viable eggs. To acknowledge the potential influence of males on number of eggs laid, we included sex as a factor in that analysis. Final brood size is the size of the brood when the larvae leave the fully consumed carcass. This measures the reduction due to culling by the parents and survival of larvae during the time on the carcass and serves as a measure of fitness per each reproductive bout. Individual offspring mass is the mass of newly eclosed adults, and it serves as a measure of individual offspring quality.

The experiment was designed as a fully crossed factorial design with two fixed factors, sex (2 levels) and carcass treatment (4 levels), and repeated measures of the response variables that corresponded to each reproductive bout (age). We evaluated the three response variables for normality of residuals and equal variances across treatment combinations. The data met these assumptions. Typically, count data such as brood size are modeled with a Poisson distribution; however, in this case the Gaussian normal distribution resulted in a more normal distribution of residuals. We used the same model for each of the three response variables: sex and carcass treatment were fixed main effects and age (measured as the chronological number of the reproductive bout) was considered a covariate. Because parents reproduced a different number of times over their lifetime (range = 2 to 6 times), age cannot be used as a main effect with a set number of levels, so we treated it as a repeated measures covariate. All possible interactions between main effects and between main effects and the covariate were included in the model. The two-way and three-way interactions that included age were of particular interest because they test for differences in the within lifetime pattern of allocation to future or current reproduction. Individual identification number was considered a random effect as a consequence of the repeated measure on the same individuals. We used the procedure GLIMMIX in SAS (SAS 9.3 SAS Institute, Cary, North Carolina, USA) for the analysis.

## Results

### Lifetime Fitness Measures

**Fig 1:**
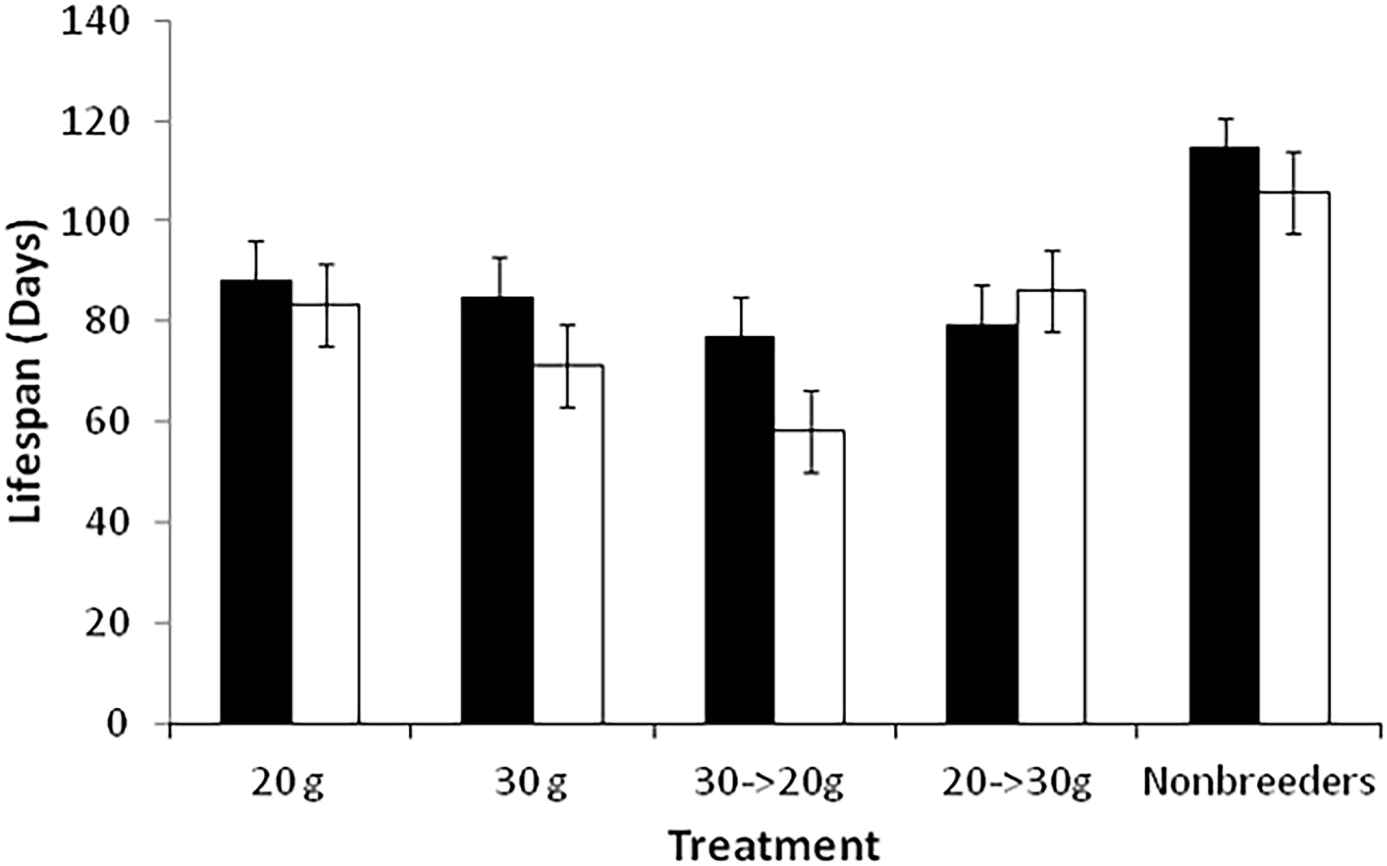
Mean (+/- 95% confidence interval) lifespan for males and females in all treatments. Males are black and females are white.

There are significant differences in life span between sexes and across treatments, and the interaction between sex and treatment is significant (Table 1). Females live an average of 80.95 days (confidence interval, hereafter CI = 77.3–84.6 days), and males live an average of 88.74 days (CI = 85.27–92.21 days). Non-breeding beetles of both sexes live significantly longer than their breeding counterparts (P < 0.0001 for all comparisons; 110 days versus 79 days, average for males and females combined across all breeding treatments). There are no differences in life span among male breeding treatments (P > 0.05 for all comparisons). However, females vary in life span depending on treatment. Females in the 20-g control treatment and the 20-g → 30-g experimental treatment have significantly longer life spans than beetles from the other two treatments (P < 0.05 for all comparisons), and females in the 30-g → 20-g experimental treatment have significantly shorter lives than all other treatments (P < 0.03 for all comparisons; Fig 1).

**Table 1:** Analysis of variance table (ANOVA) for life span and lifetime number of offspring.

Response Variable	Source	Num df/Den df	F-Value	p-value
Life Span				
	Sex	1/166	9.38	**0.0027**
	Treatment	4/166	34.95	**<.0001**
	Sex*Treatment	4/166	2.72	**0.0325**
Lifetime Number of Offspring				
	Sex	1/132	7.48	**0.0075**
	Treatment	3/132	19.89	**<.0001**
	Sex*Treatment	3/132	1.52	0.2147

**Fig 2:**
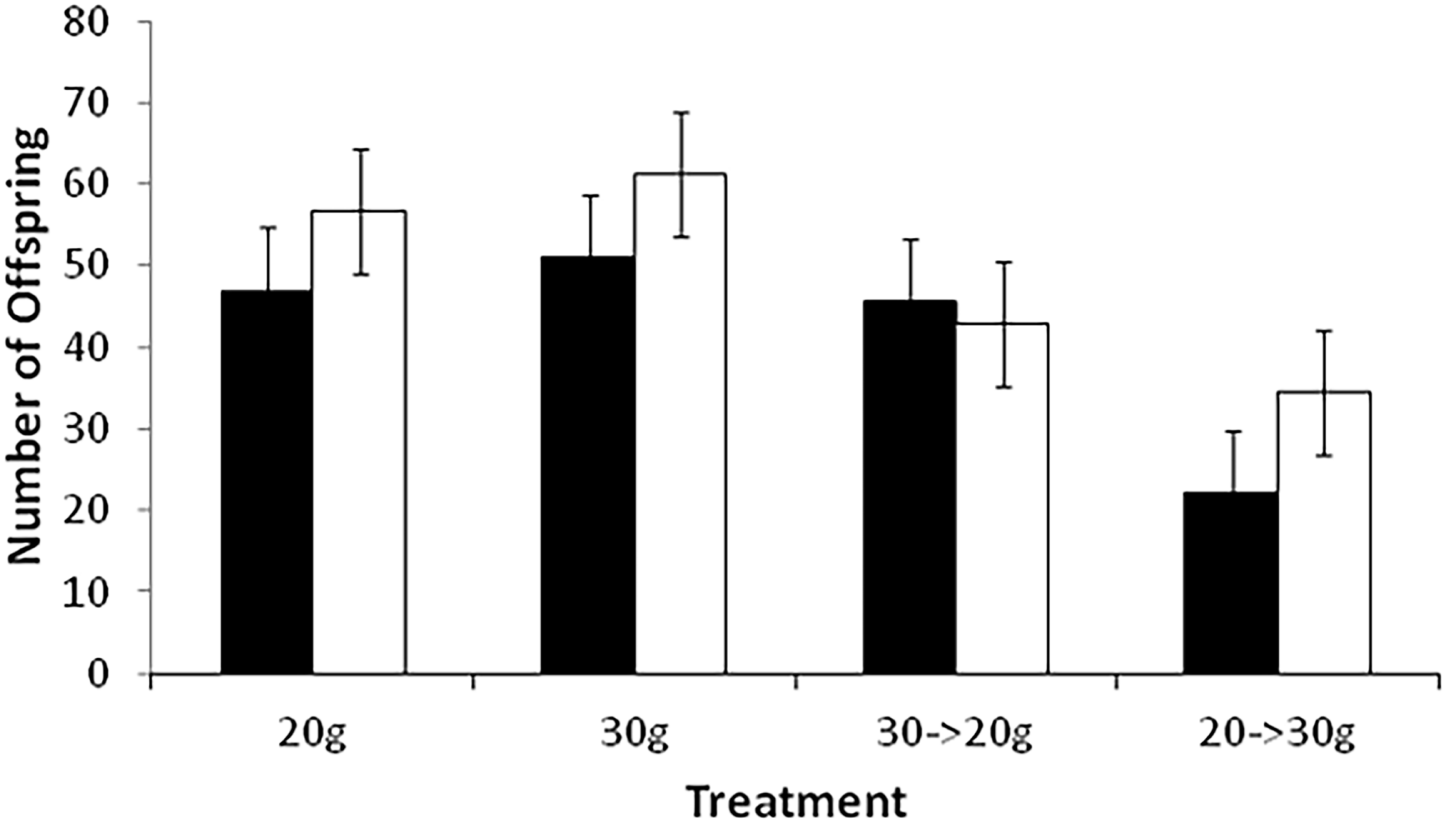
Mean (+/- 95% confidence interval) lifetime number of offspring for males and females in all reproducing treatments. Males are black and females are white.

Lifetime number of offspring differs significantly by sex and treatment, but the interaction between sex and treatment is not significant (Table 1). Females have an average of 48.9 (CI = 45.0–52.7) offspring and males have an average of 41.4 (CI = 37.5–45.2) offspring over their lifetimes (averaged across all treatments). The overallocation and underallocation treatments had lower lifetime number of offspring compared to the two control treatments (Fig 2).

### Within Lifetime Patterns

**Fig 3:**
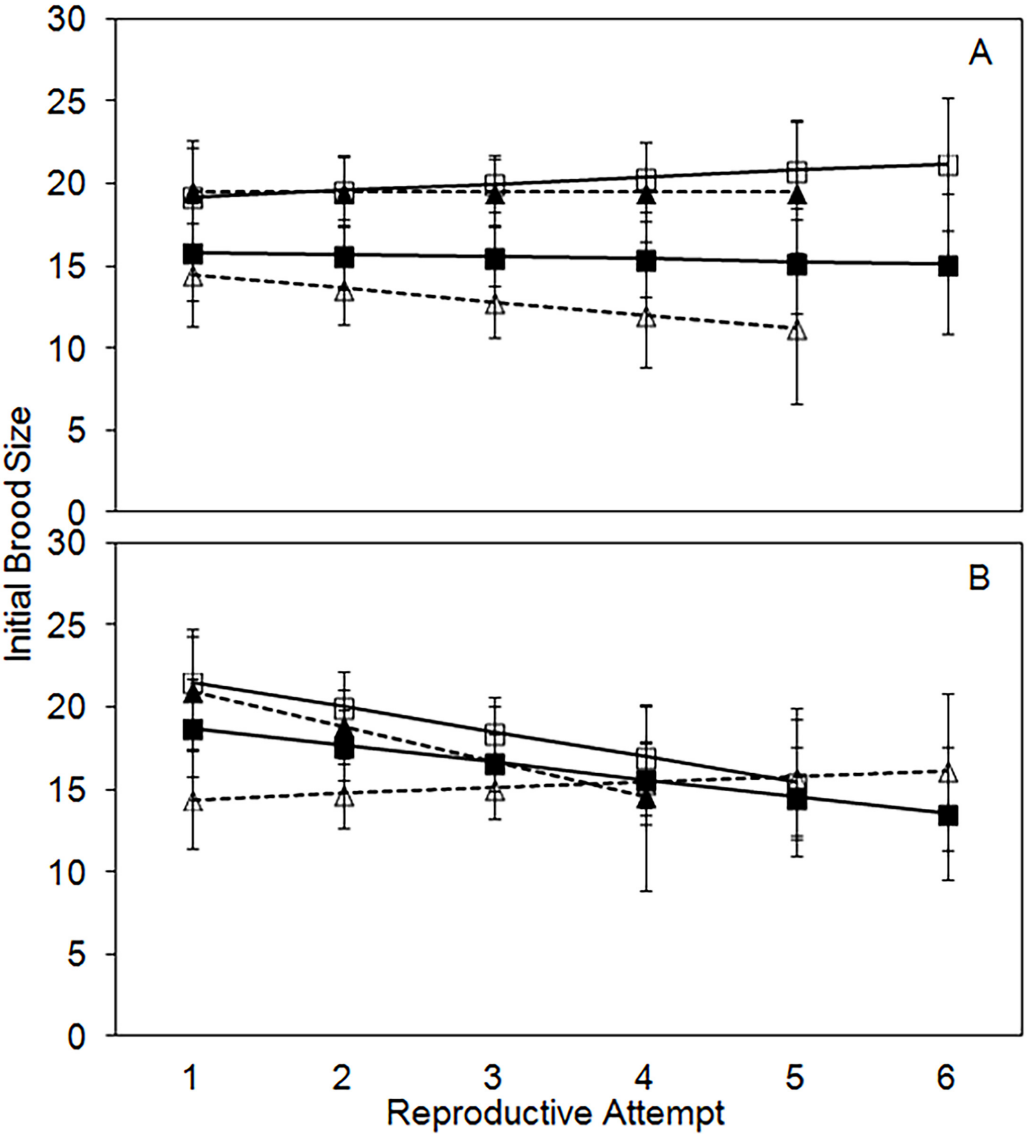
Least-squares means (+/- standard error) for initial brood size per reproductive attempt for males (A) and females (B). For both males and females the solid black line with closed squares is the 20g control treatment, the solid black line with open squares is the 30g control treatment, the dashed black line with closed triangles is the 30->20g experimental treatment, and the dashed black line with open triangles is the 20->30g experimental treatment.

Initial brood size, a measure of the number of eggs laid per each reproductive attempt, differs significantly by treatment and age, but not between sexes (Table 2). Initial brood size reflects the size of the initial carcass in each treatment. It is significantly lower in the 20-g control (16.3, CI = 15.1–17.6) and the 20-g → 30-g treatment (14.0, CI = 12.6–15.4) compared to the 30-g control (19.5, CI = 18.1–20.8) or the 30-g → 20-g treatment (18.5, CI = 16.8–20.2; all P < 0.05; Fig 3). Initial brood size is highly variable with age, but on average declines slightly through time (slope = -0.8; Fig 3).

**Table 2:** Analysis of covariance table for initial brood size, final brood size, and mean offspring size for each reproductive attempt. Age reflects the number of reproductive attempt and is considered a covariate.

Response Variable	Effect	Num df/Den df	F-Value	p-value
Initial Brood Size				
	Sex	1/134.8	2.66	0.1050
	Treatment	3/131.3	3.74	**0.0128**
	Age	1/293.5	4.46	**0.0356**
	Sex*Treatment	3/131.3	0.68	0.5648
	Age*Sex	1/293.5	2.67	0.1033
	Age*Treatment	3/259.8	0.27	0.8469
	Age*Sex*Treatment	3/259.8	1.74	0.1598
Final Brood Size				
	Sex	1/350.7	20.26	**<.0001**
	Treatment	3/349.5	8.79	**<.0001**
	Age	1/302.2	13.13	**0.0003**
	Sex*Treatment	3/349.5	3.74	**0.0115**
	Age*Sex	1/302.2	8.69	**0.0034**
	Age*Treatment	3/301.9	0.62	0.6035
	Age*Sex*Treatment	3/301.9	4.60	**0.0036**
Mean Offspring Mass				
	Sex	1/367	0.46	0.4992
	Treatment	3/367	5.13	**0.0024**
	Age	1/367	0.34	0.5582
	Sex*Treatment	3/367	1.81	0.1502
	Age*Sex	1/367	1.43	0.2334
	Age*Treatment	3/367	0.07	0.9737
	Age*Sex*Treatment	3/367	2.72	**0.0458**

**Fig 4:**
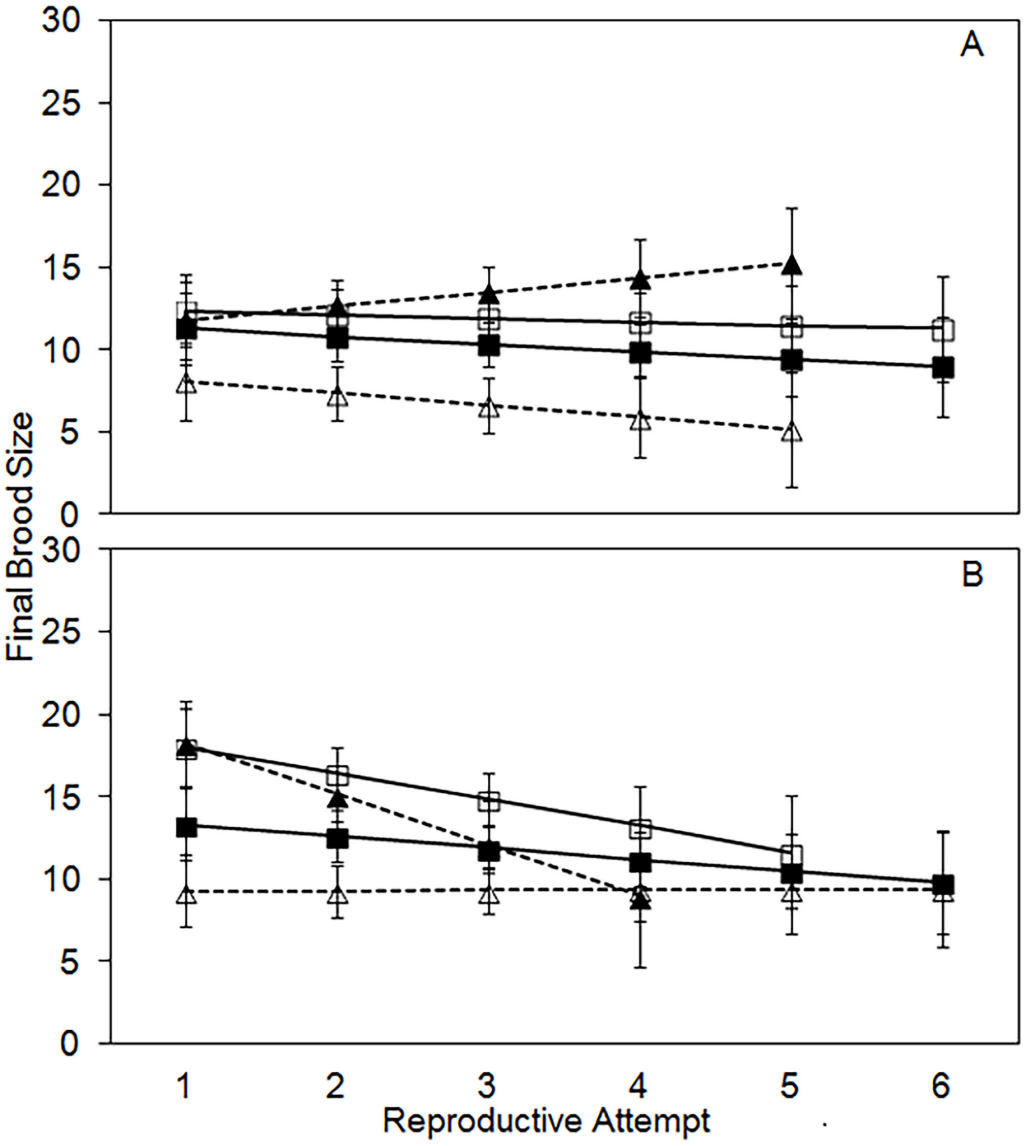
Least-squares means (+/- standard error) for final brood sizes per reproductive attempt for males (A) and females (B). For both males and females the solid black line with closed squares is the 20g control treatment, the solid black line with open squares is the 30g control treatment, the dashed black line with closed triangles is the 30->20g experimental treatment, and the dashed black line with open triangles is the 20->30g experimental treatment.

Final brood size, the number of eclosed adult offspring for each reproductive attempt, differs significantly by sex, treatment, age, an interaction between sex and treatment, an interaction between age and sex, and the three-way interaction between age, sex, and treatment (Table 2). Final brood size for females (12.5, CI = 11.7–13.3) is higher than for males (10.6, CI = 9.9–11.4). Similar to initial brood size, final brood size somewhat reflects the initial carcass size. It is significantly lower in the 20-g control (11.3, CI = 10.3–12.3) and the 20-g → 30-g treatment (8.1, CI = 7.0–9.2) compared to the 30-g control (13.7, CI = 12.7–14.7) or the 30-g → 20-g treatment (13.2, CI = 11.9–14.5; all P < 0.02). Males and females respond differently to the carcass treatments through time. In males, final brood size does not differ significantly through time for the two control treatments and the 30-g → 20-g experimental, but it declines through time for the 20-g → 30-g experimental treatment (Fig 4A). In females, final brood size decreases significantly through time for the two control treatments and the 30-g → 20-g experimental treatment, but it does not differ through time in the 20-g → 30-g experimental treatment (Fig 4B).

**Fig 5:**
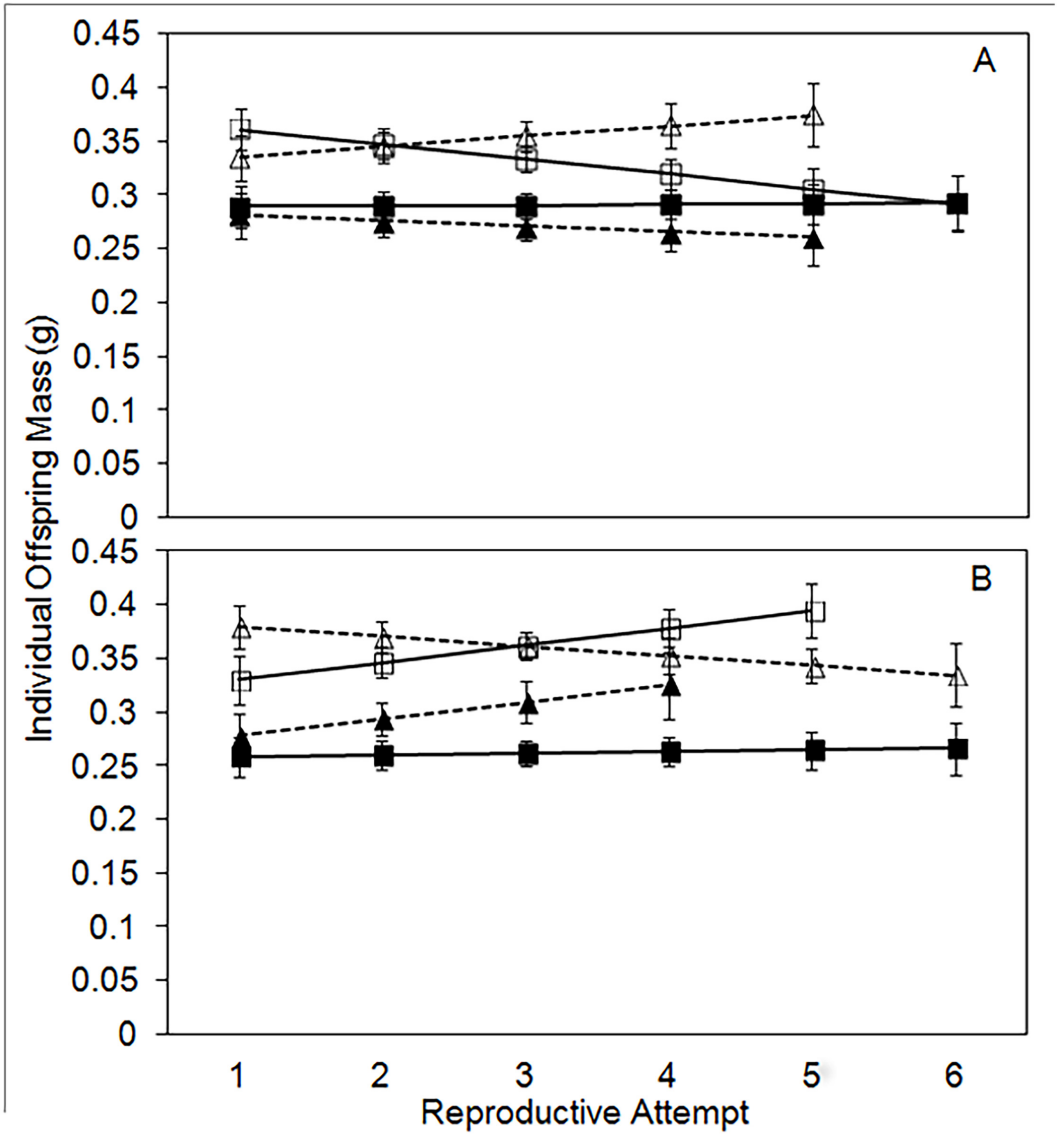
Least-squares means (+/- standard error) for mean offspring mass per reproductive attempt for males (A) and females (B). For both males and females the solid black line with closed squares is the 20g control treatment, the solid black line with open squares is the 30g control treatment, the dashed black line with closed triangles is the 30->20g experimental treatment, and the dashed black line with open triangles is the 20->30g experimental treatment.

Mean individual offspring size per reproductive attempt differs significantly by treatment and a three-way interaction between age, sex, and treatment (Table 2). Offspring size reflects final carcass size in all treatments. Mean individual offspring size on 20-g carcasses is 0.28-g (CI = 0.26–0.29-g), on 30-g carcasses is 0.35-g (CI = 0.33–0.36-g), on experimental 30-g → 20-g carcasses is 0.29-g (CI = 0.27–0.31-g), and on experimental 20-g → 30-g carcasses is 0.36-g (CI = 0.34–0.38-g). Males and females respond differently to the 30-g carcass treatment through time. In males, individual offspring mass in the 30-g control treatment declines significantly through time (Fig 5A); whereas, in females, individual offspring mass in the 30-g control treatment increases significantly through time (Fig 5B). In both males and females, average individual offspring mass remains constant through time in the other three treatments.

## Discussion

Male and female *N*. *orbicollis* take very different approaches to parental care. The key difference between the sexes that generates many of the other observed patterns is the difference in number of offspring kept on a carcass after culling. Females keep substantially more offspring than males on a given size carcass. Brood sizes resulting from female culling match resource availability, such that larger broods are found on larger carcasses (Fig 4B; difference in final brood sizes between 20-g and 30-g carcasses). In contrast, brood sizes resulting from male culling do not differ with carcass size (Fig 4A; no difference between final brood sizes on 20-g and 30-g carcasses), but rather reflect a relatively constant 60% of initial brood size. As a consequence, total mass of offspring produced per reproductive bout and over a lifetime is lower in males than females. This difference between the sexes suggests that sexual conflict over brood size could be an important component of the burying beetle breeding system.

Our results differ from those of Walling et al. [25] who found no difference in brood size between the sexes in *N*. *vespilloides*. These contrasting results could be a result of species-specific differences or a result of differences in experimental design. Whereas we used carcass sizes that normally result in brood regulation through filial cannibalism in *N*. *orbicollis*, Walling et al. [25] focused on carcass sizes beyond which *N*. *vespilloides* regulates brood size [33]. As a result, brood sizes may have been influenced more by female fecundity than active regulation through cannibalism.

Why do males keep fewer offspring on a carcass compared to females? We consider three potential explanations. First, males may be selected to favor a different balance between offspring number and individual offspring size. Body size is an important determinant of fitness in burying beetles with larger individuals being more successful in competition for a carcass [22]. Theoretically, males could increase their lifetime fitness by producing fewer, but larger offspring (i.e. higher quality offspring) relative to other males that kept larger broods. However, although males keep fewer offspring on a given-sized carcass relative to females, their offspring did not differ in size compared to females (Table 2 and Fig 5). Instead, males keep fewer offspring on a given carcass size and experience reduced fitness by our measure (lifetime number of offspring).

Second, differences in culling between sexes could result from differences in lifetime strategy for solving the tradeoff between current and future reproduction. Females raising offspring on smaller carcasses adopt a conservative lifetime strategy by keeping fewer offspring and using more of the carcass for their own energy reserves [5,32]. As a result, females breeding on smaller carcasses have longer lifespans and more reproductive attempts compared to females breeding on larger carcasses, and thus females on large and small carcasses have equal fitness [5]. Theoretically, males could be taking a similar approach by keeping fewer offspring on a given carcass, and therefore survive for more reproductive bouts compared to females. Thus, differential culling may be a result of differences between the sexes in the balance between current and future reproduction. However, males do not experience extended lifespans compared to females (Fig 1). Males do not appear to be using a different strategy for balancing current versus future reproduction.

Third, an as yet untested possible explanation for why males keep fewer offspring than females is for paternity assurance. Female burying beetles store sperm from previous matings [34], which they use to fertilize eggs along with the sperm of the resident male [35]. After a carcass is buried, satellite males attempt extra-pair copulations with the resident female and, if successful, tend to copulate with females for longer periods of time than resident males [36]. In *N*. *vespilloides*, offspring from female brood parasites tend to arrive on a carcass first, and resident females use a time-dependent cue while culling the brood to increase the proportion of the brood that is their own [37–38]. By some similar rule, resident males may differentially cull offspring to increase their paternity. Culling of more offspring by males compared to females may have little to do with reproductive strategy of offspring size or cost of reproduction, but rather it may represent an evolved response to the risk of extra pair paternity.

Largely as a consequence of keeping different numbers of offspring on a carcass, male and female *N*. *orbicollis* experience costs of reproduction differently. Females change brood size and their corresponding level of investment proportional to resource availability (Fig 4B). Overallocation (30-g → 20-g) is costly for females and results in a dramatic decrease in lifetime fitness (Fig 2) especially compared to reproduction on 30-g carcasses. Underallocation (20-g → 30-g) allows females to experience a longer reproductive lifespan compared to the overallocation treatment. However, underallocation results in decreased fitness for females because final brood sizes are reduced compared to 20-g controls (Fig 4), possibly from lack of ability to maintain the larger carcass with a smaller brood. Taken together these results suggest that the cost of reproduction for females depends on resource availability and acts as a constraint on female reproductive investment.

In contrast to patterns of cost of reproduction in females, males do not experience costs of reproduction associated with resource availability because they keep far fewer offspring on a given carcass. Final number of offspring per brood differs much less on large and small carcasses compared to females, and the effect of overallocation on males is negligible in terms of lifespan and lifetime number of offspring. Fewer offspring kept on a carcass enhanced the negative effect of underallocation for males such that the number of offspring was lower even than the normal brood size for a 20-g carcass. Like females, males may have difficulty controlling decomposition of the larger carcass with a small broods. Taken together these results suggest that reproductive strategy of males does not change depending on the amount of available resources. Males incurred the same costs of reproduction in all treatments.

Both males and females experience decreased lifespans as a cost of reproduction, consistent with results from several other insect species [8,14,39–42]. However, specific activities that produce the cost of reproduction vary among species and sexes. Male dung beetles (*O*. *binodis*) incur a cost of reproduction manifest in lifespan from mating and courtship, but females showed no effects of these activities compared to non-reproducing individuals [14]. Lifespan was reduced due to egg production and parental care in female dung beetles of a different species [8]. A similar result was found in seed beetles (*Callosobruchus maculatus*) where males suffered from reduced lifespan due to mating [41], while females suffered from reduced lifespan due to egg-laying [40]. Although reproduction generally reduces lifespan for males and females, the source and magnitude of the effect differs between the sexes. Male reproductive costs are typically fixed relative to resource availability; whereas, female costs of reproduction vary based on resource availability consistent with our results in *N*. *orbicollis*.

Terminal investment has been demonstrated in female burying beetles [5], but can males terminally invest in a system where offspring number and size are not strongly related to resource availability? For males, the tradeoff between current and future reproduction is not as tightly linked as in females (because they cull offspring below the level a carcass can support). In such a system, males cannot increase current reproductive output by limiting allocation to self, so terminal investment may not be compatible with the level of culling provided by males. Older males could provide better post-hatching parental care [43], and thus produce larger offspring similar to the pattern seen in females. However, males appear to be poorer at parental care as they age, and offspring size declines. Our data do not support the presence of male terminal investment in *N*. *orbicollis*.

Our experimental approach of using uniparental parents to evaluate life history traits in a biparental species is contingent on the level of investment being similar between the two types of care [18]. If uniparental parents increase their level of investment to compensate for the loss of their partner, then our experiment would overestimate the level of investment. Female burying beetles do not compensate for the loss of mate during the period of care we focused on but males do [44]. As a result, we potentially could be overestimating male but not female parental investment. Given that males were unaffected by our brood manipulation and do not seem to be resource limited, it is highly unlikely that any compensation by male parents compromises our conclusions.

Large-scale experiments such as this always represent a tradeoff between logistical constraints and the number of treatments required in a large factorial design. In this case, we did not have time ore space to replicate the uniparental female treatments along with the same treatments in uniparental males. To make the comparison between the sexes we used data from a study done few years earlier [5]. We went to great lengths to create exactly the same experimental conditions, both experiments were done in the same location, we used beetles collected from the same source population, and we used F1 and F2 generation beetles to avoid influences from the field population. Also, one of the authors oversaw both experiments to ensure the same protocols were used. To the extent possible, other than doing these two experiments at the same time, all other conditions were identical. We realize that the observed differences between males and females may be partially due to random variation in the sample, or small unobservable differences among individuals collected at two different times. The main difference we identify in this paper is a difference in the number of offspring culled by each sex. Males cull about 40% of offspring on average, and female culling, as noted above, differs by carcass size but averages about 20%. This difference is relatively large, and thus is likely to reflect real differences between sexes and not small differences in timing of experiments. As with all large-scale experiments, reproducibility of the results can only be determined by redoing the experiment multiple times.

## Conclusions

Female *N*. *orbicollis* incur different costs of reproduction depending on the amount of resources available because they alter their reproductive tactic in a way that maximizes their fitness. However, males invest a similar amount of effort into each brood, regardless of resource availability, which leads to a decrease in their fitness in comparison to females. Further investigation is necessary to determine the evolutionary cause of biparental care in this species. To determine why the cost of reproduction differs between the sexes in this biparental species will require further investigation into the evolution of biparental care.
